# A centrality measure for cycles and subgraphs II

**DOI:** 10.1007/s41109-018-0064-5

**Published:** 2018-06-08

**Authors:** Pierre-Louis Giscard, Richard C. Wilson

**Affiliations:** Department of Computer Science, University of York, Deramore Lane, Heslington, York, YO10 5GH UK

**Keywords:** Centrality of groups of nodes, Protein complexes, Eigenvector centrality, Group-centrality

## Abstract

In a recent work we introduced a measure of importance for groups of vertices in a complex network. This centrality for groups is always between 0 and 1 and induces the eigenvector centrality over vertices. Furthermore, its value over any group is the fraction of all network flows intercepted by this group. Here we provide the rigorous mathematical constructions underpinning these results via a semi-commutative extension of a number theoretic sieve. We then established further relations between the eigenvector centrality and the centrality proposed here, showing that the latter is a proper extension of the former to groups of nodes. We finish by comparing the centrality proposed here with the notion of group-centrality introduced by Everett and Borgatti on two real-world networks: the Wolfe’s dataset and the protein-protein interaction network of the yeast *Saccharomyces cerevisiae*. In this latter case, we demonstrate that the centrality is able to distinguish protein complexes

## Introduction

### Context

In our previous work on the subject, we argued the need to go beyond vertices when analysing complex networks. In fact, remarks to this end can be found scattered in the literature (Contreras and Fagiolo 2014; Estrada and Rodríguez-Velázquez 2005; Milo and et al 2002;Mukhtar and et al 2011;Yeger-Lotem and et al 2004). For example, studies of gene regulatory networks have shown that “motif-based centralities outperforms other methods” and can discern interesting network features not grasped by more traditional vertex centralities (Koschützki et al. 2007;Koschützki and Schreiber 2008). Another example is provided by the notion of protein essentiality, a property now understood to be determined at the level of protein complexes, that is groups of proteins in the protein-protein interaction network (PPI) rather than at the level of individual proteins (Hart et al. 2007;Ryan et al. 2013). In addition, further biological properties have been tied to ensembles of genes or proteins, e.g. the notion of synthetic lethality, where the simultaneous deactivation of two genes is lethal while the separate deactivation of each is not (Nijman 2011). Since measures of importance for nodes constitute a key tool in the study of complex networks, it is only logical to expect that similar tools for ranking groups of vertices could find widespread applications throughout network analysis.

In this spirit, we proposed in (Giscard and Wilson 2017b) a measure of importance for groups of nodes (henceforth called “subgraphs"), that has the following desirable properties:

1. Provided the edge weights are non-negative, the centrality *c*(*H*) of a subgraph *H* is always between 0 and 1.
2. The precise value *c*(*H*) taken by the centrality on a subgraph *H* is the fraction of all network flows intercepted by *H*.
3. For subgraphs comprising a single node *H*≡{*i*}, the centrality measure *c*({*i*}) yields the same ranking than the eigenvector centrality. In other terms, it induces the eigenvector centrality over vertices.
4. Computationally, *c*(*H*) costs no more to compute per subgraph *H* than ordinary vertex-centralities. What is computationally costly however, is to compute it over all subgraphs.

In (Giscard and Wilson 2017b), we have shown, by analysing real-world networks from econometry and biology, that *c*(.) performs better than centralities defined from naive sums of vertex-centralities. Concretely, we demonstrated that subgraph centralities defined from sums of the resolvent, exponential and eigenvector centralities failed to account for even the dominant events affecting input-output economic networks. In biology, we used *c*(.) to construct a model of protein-targeting by pathogens that achieved a 25% improvement over the state of the art one.^1^

In this work, we establish further properties of the centrality measure *c*(.) and present its rigorous mathematical underpinnings. We also compare this centrality with the notion of group-centrality presented by Everett and Borgatti in (Everett and Borgatti 1999) on real-world networks.

### Notations and centrality definition

The measure of cycle and subgraph centrality we propose is rooted in recent advances in the algebraic combinatorics of walks on graphs. Here we only define the few concepts from this background that are necessary to comprehend the centrality measure.

We consider a finite network $G = \left (\mathcal {V} ;\mathcal {E}\right)$ with $N=|\mathcal {V}|$ nodes and $M=|\mathcal {E}|$ edges and which may be weighted and directed. The adjacency matrix of *G* is denoted A_*G*_ or simply A. If *G* is weighted then the entry A_*ij*_ is the weight of the edge *e*_*ij*_ from *i* to *j* if this edge exists, and 0 otherwise.

A *induced subgraphH* of *G*, also called simply a *subgraph* of *G* and denoted *H*≺*G*, is a set of vertices $ \mathcal {V}_{H}\subseteq \mathcal {V}$ together with the set of all edges linking these vertices in *G*, $\mathcal {E}_{H}=\{e_{ij}\in \mathcal {E}:\,i,j\in \mathcal {V}_{H}\}$. The subgraphs considered in this article are not necessarily connected.

A *walkw* of length *ℓ*(*w*) from *v*_*i*_ to *v*_*j*_ on *G* is a sequence $w = e_{i i_{1}} e_{i_{1} i_{2}} \cdots e_{i_{\ell -1} j}$ of *ℓ* contiguous edges. The walk *w* is *open* if *i*≠*j* and *closed* otherwise.

A *simple cycle*, also known in the literature under the names *loop*, *cycle*, *elementary circuit* and *self-avoiding polygon*, is a closed walk $w = e_{i i_{1}} e_{i_{1} i_{2}} \cdots e_{i_{\ell -1} i}$ which does not cross the same vertex twice, that is, the indices *i*,*i*_1_,…,*i*_*ℓ*−1_ are all different.

We now recall the definition of the centrality for cycles and subgraphs, introduced in (Giscard and Wilson 2017b).

#### Definition 1.

[Centrality] Let *G* be a (weighted di)graph and let A be the adjacency matrix of *G*, including weights if any. Define *λ* the maximum eigenvalue of A. For any cycle *γ*, let A_*G*∖*γ*_ be the adjacency matrix of the graph *G* where all vertices visited by *γ* and the edges adjacent to them have been removed. Then we define the centrality *c*(*γ*) of the cycle *γ* as

$$c(\gamma):=\det\left(\mathsf{I}-\frac{1}{\lambda}\mathsf{A}_{G\backslash \gamma}\right). $$

More generally, for any non-empty subgraph *H* of *G*, we define the centrality of *H* as

$$c(H):=\det\left(\mathsf{I}-\frac{1}{\lambda}\mathsf{A}_{G\backslash H}\right). $$

**Fig. 1:**
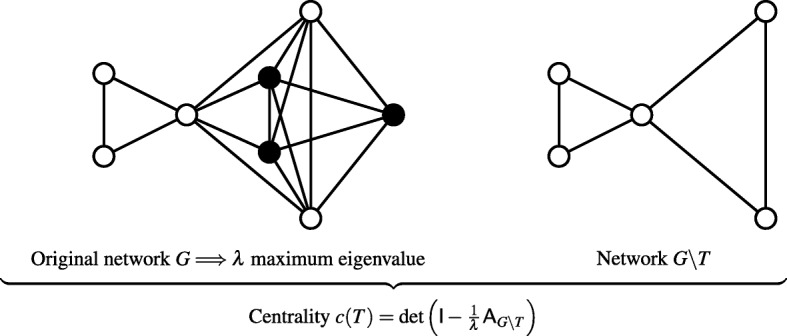
Schematic representation of the calculation of a centrality. Left : full network *G*, with in black the group of three vertices forming a triangle *T*, the centrality of which is desired. Right: graph *G*∖*T* where all vertices belonging to *T* have been removed. The matrix A_*G*∖*T*_ is the adjacency matrix of *G*∖*T*

The calculation of the centrality is illustrated on Fig. 1.

As we have shown in (Giscard and Wilson 2017b), these centralities not only reflect the relative importance of cycles or subgraphs, but their values have a precise meaning too. Indeed, *c*(*H*) is the fraction of all information flows on the network that are intercepted by the subgraph *H*. As such, and as long as the network has no negative edge-weights, the centrality is always between 0 and 1, which is numerically advantageous,

$$0\leq c(H)\leq 1. $$

Because it has a concrete real-world meaning as fraction of network flows, the value of the centrality can be assessed with respect to external informations when available. More generally, it enriches the analysis in that it does not only produce a ranking of groups of nodes, but it also quantitatively ties these groups’ importance with an immediately meaningful quantity, e.g. a fraction of capital flow, of successions of proteins interactions or of social interactions depending on the context.

It the following section we give the full, rigorous mathematical proof of the main theorem underpinning these results and which relates the centrality *c*(*γ*) of a cycle *γ* with network flows. This theorem was presented as Proposition 1 in (Giscard and Wilson 2017b) but was only given a qualitative proof there, owing to length constraints. Note, we focus on the centrality of simple cycles as it is precisely in this context that the rigorous proof appears as an extension of a number theoretic sieve. The case of arbitrary subgraphs is similar, and we operate with no loss of generality.

## Centrality and network flows: a rigorous mathematical proof

We first need to recall some combinatorial notions introduced in the context of the extension of number theory satisfied by walks on graphs (Giscard and Rochet 2017). The central objects of this earlier study are *hikes*, a hike *h* being an unordered collection of disjoint closed walks. Hikes can be also be seen as equivalence classes on words $W=\gamma _{i_{1}}\gamma _{i_{2}}\cdots \gamma _{i_{n}}$ over the alphabet of simple cycles *γ*_*i*_ of a graph. Two words *W* and *W*^′^ are equivalent if and only if *W*^′^ can be obtained from *W* through allowed permutations of consecutive simple cycles. In this context, two simple cycles are allowed to commute if and only if they are vertex disjoint $\mathcal {V}(\gamma _{i})\cap \mathcal {V}(\gamma _{j})=\emptyset \iff \gamma _{i}\gamma _{j}=\gamma _{j}\gamma _{i}$.

For example, if *γ*_1_ and *γ*_2_ commute but neither commute with *γ*_3_, then *γ*_1_*γ*_2_ and *γ*_2_*γ*_1_ represent the same hike, but *γ*_1_*γ*_3_*γ*_2_ and *γ*_2_*γ*_3_*γ*_1_ are distinct hikes.

The letters $\gamma _{i_{1}},\cdots, \gamma _{i_{n}}$ found in a hike *h* are called its prime divisors. This terminology is due to the observation that simple cycles obey the defining property of prime elements in the semi-commutative monoid $\mathcal {H}$ of hikes. In addition, they constitute the formal, semi-commutative, extension of prime numbers (Giscard and Rochet 2017).

Two special types of hikes will be important for our purpose here:

A *self-avoiding hike* is a hike all prime factors of which commute with one another. In other terms, it is collection of vertex-disjoint simple cycles.

A walk, defined earlier in section Notations and centrality definition, can be shown to be hikes with a unique right prime divisor (Giscard and Rochet 2017), a characterisation which is both necessary and sufficient so that any hike with a unique right prime divisor is a walk.

It may perhaps help the reader’s intuition to know that in the extension of number theory satisfied by hikes, hikes are the extension of the integers, self-avoiding hikes are the square-free integers and walks are integers of the form *p*^*k*^, with *p* prime and $k\in \mathbb {N}$.

Now we claim that the centrality *c*(*γ*) of a simple cycle *γ* is exactly the fraction of all hikes *h* (including infinite length ones) such that all right prime divisors of *h* intercept *γ*, that is no right prime divisor of *h* is vertex-disjoint with *γ* and commutes with it. This later observation implies that *γ* is the only right prime divisor of *hγ*. Thus, the claim we make is equivalent to stating that *c*(*γ*) is the proportion of all hikes *h* such that *hγ* is a walk.

### Theorem 1.

Let *G* be a finite (di)graph with adjacency matrix A and let *γ* be a simple cycle on *G*. Then the total number *n*_*γ*_(*k*) of closed walks of length *k* on *G* with right prime divisor *γ* is asymptotically equal to

$$n_{\gamma}(k)\sim c(\gamma)\!\left(\frac{1}{\det\left(\mathsf{I}-z\mathsf{A}\right)}\right)\![k],~~\text{as}~~k\to\infty, $$

where (1/ det(I−*z*A))[*k*] stands for the coefficient of order *k* in the series 1/ det(I−*z*A).

### *Proof*

The proof relies on a very general combinatorial sieve. Let $\mathcal{H}_{\ell}:=\{h\in \mathcal{H} :~\ell (h)=\ell \}$ be the set of hikes of length *ℓ*, $\mathcal {P}\subsetneq \mathcal {H}$ be a set of primes and $\mathcal {P}^{\text {s.a.}}$ the set of all self-avoiding hikes constructible from $\mathcal {P}$. Let $S(\mathcal {H}_{\ell },\mathcal {P})$ be the number of hikes in $\mathcal {H}_{\ell }$ which are not right-divisible by any prime of $\mathcal {P}$. The semi-commutative extension of the sieve of Erathostenes-Legendre yields

$$S(\mathcal{H}_{\ell},\mathcal{P}) = \sum_{d\in \mathcal{P}^{\text{s.a.}}} \mu(d) |\mathcal{M}_{d}|, $$

with $|\mathcal {M}_{d}|$ the number of multiples of *d* in $\mathcal {H}_{\ell }$. Furthermore, *μ*(*d*) is the Möbius function on hikes, which is (Giscard and Rochet 2017)

$$\mu(h)=\left\{\begin{array}{ll} (-1)^{\Omega(h)},&\text{if {h} is self-avoiding,}\\ 0,&\text{otherwise,} \end{array}\right. $$

where *Ω*(*h*) is the number of prime divisors of *h*, including multiplicity. □

In order to progress, we seek a multiplicative function prob(.) such that $|\mathcal {M}_{d}| = \text {prob}(d)|\mathcal {H}_{\ell }| + r(d)$, $|\mathcal {H}_{\ell }|:=\text {card}(\mathcal {H}_{\ell })$. In this expression, prob(*d*) approximates the probability that a hike taken uniformly at random in $\mathcal {H}_{\ell }$ is right-divisible by *d*. If edge-weights are present, the hikes are not all uniformly probable but follow a distribution dependent on these weights. In any case, no knowledge of this distribution is required here and the meaning of prob(.) is only mentioned to help the reader understanding. Similarly, $m(d)=\text {prob}(d)|\mathcal {H}_{\ell }|$ is the expected number of multiples of *d* in $\mathcal {H}_{\ell }$. Finally, *r*(*d*) is the associated error term, arising from the fact that $|\mathcal {M}_{d}|$ is not truly multiplicative. Supposing that we can identify the *m*(.) function, we would obtain

$$S(\mathcal{H}_{\ell},\mathcal{P}) = \sum_{d\in \mathcal{P}^{\text{s.a.}}} \mu(d) m(d) + \sum_{d\in \mathcal{P}^{\text{s.a.}}} \mu(d) r(d). $$

Contrary to number theory, the first term does not admit any simpler form without further assumptions on $\mathcal {P}$. This is because of the possible lack of commutativity between some elements of $\mathcal {P}$. We note however that since *μ*(*d*) is non-zero if and only if *d* is self-avoiding, and since we have required that *m*(.) be multiplicative,^2^ then it follows that the first term is entirely determined from the values of *m*(.) over the primes of $\mathcal {P}$.

We therefore turn to determining *m*(*γ*) for *γ* prime. The set of left-multiples of *γ* in is {*hγ*, *h*∈}, which is in bijection with the set {*h*∈, *ℓ*(*h*)≥*ℓ*(*γ*)}. Thus, the number of left-multiples of *γ* in $\mathcal {H}_{\ell }$, is exactly $|\mathcal {H}_{\ell -\ell (\gamma)}|$. Then

$$\text{prob}(\gamma) + \frac{r(\gamma)}{|\mathcal{H}_{\ell}|} = \frac{|\mathcal{H}_{\ell-\ell(\gamma)}|}{|\mathcal{H}_{\ell}|}. $$

Seeking the best possible probability function prob(*γ*), let us suppose that once this function has been chosen, the error term of the above equation vanishes in the limit *ℓ*→*∞*. If this is true, then we obtain

$$\text{prob}(\gamma) = {\lim}_{\ell\to \infty}\frac{|\mathcal{H}_{\ell-\ell(\gamma)}|}{|\mathcal{H}_{\ell}|}. $$

In order to progress, we make an important observation regarding the cardinality of the set $\mathcal {H}_{\ell }$:

### Lemma 1.

Let *G* be a finite (directed) graph. Let $\mathcal {H}_{\ell }:=\{h\in \mathcal {H}:~\ell (h) = \ell \}$ be set of all hikes on *G* of length *ℓ*. Then, there exists $\Lambda \in \mathbb {R}^{+}$ and a bounded function $f:\mathbb {N}\mapsto \mathbb {R}$ such that ${\lim }_{\ell \to \infty }f(\ell)$ exists and for $\ell \in \mathbb {N}^{*}$ we have exactly

$$|\mathcal{H}_{\ell}|=\Lambda^{\ell} f(\ell). $$

If the absolute value of the largest eigenvalue *λ* of *G* has multiplicity *g*, then *Λ*=*λ*^*g*^.

### *Proof*

This follows directly from the ordinary zeta function on hikes *ζ*(*z*)= det(I−*z*A)^−1^, from which we have

$$|\mathcal{H}_{\ell}|=\left(\frac{1}{\det\left(\mathsf{I}-z\mathsf{A}\right)}\right)\![\ell] =\sum_{i_{1},\cdots,\, i_{N}\vdash \ell} \lambda^{i_{1}}_{1}\lambda^{i_{2}}_{2}\cdots \lambda^{i_{N}}_{N}=\lambda^{\ell}\!\! \sum_{i_{1},\cdots,\, i_{N}\vdash \ell} \lambda^{i_{1}-\ell}\lambda^{i_{2}}_{2}\cdots \lambda^{i_{N}}_{N} $$

where the sums run over all positive values of *i*_*j*_≥0 such that $\sum _{j} i_{j} = \ell $ and *λ*≡*λ*_1_ is the eigenvalue of the graph with the largest absolute value. We assume for the moment that *λ* is unique and let $ f(\ell):= \sum _{i_{1},\cdots, \,i_{N}\vdash \ell } \lambda ^{i_{1}-\ell }\lambda ^{i_{2}}_{2}\cdots \lambda ^{i_{N}}_{N}. $ This function is clearly bounded and

$${\lim}_{\ell\to\infty} f(\ell) = {\lim}_{z\to\lambda^{-1}}(1-z\lambda)\zeta(z), $$

exists and is finite. If |*λ*| is not unique and has multiplicity *g*, then one should pick *λ*^*g*^ for the scaling constant together with *f*(*ℓ*)=*ζ*(*z*)[*ℓ*]*λ*^−*gℓ*^. In all cases the Lemma follows. □

Proceeding with the result of Lemma 1 and assuming that the largest eigenvalue is unique for simplicity, the existence of the limit for *f* gives

$$\text{prob}(\gamma) = {\lim}_{\ell\to \infty}\frac{\lambda^{\ell-\ell(\gamma)} f\left(\ell-\ell(\gamma)\right)}{\lambda^{\ell} f(\ell)} = \lambda^{-\ell(\gamma)}. $$

The prob(.) function is multiplicative over the primes–recall these are the simple cycles–as desired. It yields $m(\gamma) = |\mathcal {H}_{\ell }| \lambda ^{-\ell (\gamma)}$ and the associated error term is

$$\begin{array}{*{20}l} r(\gamma) = |\mathcal{H}_{\ell-\ell(\gamma)}| - |\mathcal{H}_{\ell}| \lambda^{-\ell(\gamma)} &= \lambda^{\ell-\ell(\gamma)}\left(f\left(\ell-\ell(\gamma)\right)-f(\ell)\right). \end{array} $$

To establish the validity of these results, we need only verify that they are consistent with our initial assumption concerning the error term, namely that $r(\gamma)/|\mathcal {H}_{\ell }|$ vanishes in the limit *ℓ*→*∞*. The existence of the limit of *f* implies ${\lim }_{\ell \to \infty }|f\left (\ell -\ell (\gamma)\right)-f(\ell)|=0$ and therefore that

$${\lim}_{\ell\to\infty }\frac{r(\gamma)}{ |\mathcal{H}_{\ell}|}={\lim}_{\ell\to\infty }\,\lambda^{-\ell(\gamma)}\left(f\left(\ell-\ell(\gamma)\right)-f(\ell)\right) =0, $$

as required.

We are now ready to proceed with general self-avoiding hikes. Let *d*=*γ*_1_⋯*γ*_*q*_ be self-avoiding. Then since *m* is multiplicative and the length is totally additive over $\mathcal {H}$, $m(d) = \prod _{i} m(\gamma _{i}) = \lambda ^{-\sum _{i} \ell (\gamma _{i})} = \lambda ^{-\ell (d)}$. The associated error term follows as

$$r(d) = |\mathcal{H}_{\ell-\ell(d)}| - |\mathcal{H}_{\ell}| \lambda^{-\ell(d)} = \lambda^{\ell-\ell(d)}\left(f\left(\ell-\ell(d)\right)-f(\ell)\right). $$

Inserting these forms for *m*(*d*) and *r*(*d*) in the semi-commutative Erathostenes-Legendre sieve yields the sieve formula

$$S(\mathcal{H}_{\ell},\mathcal{P}) = |\mathcal{H}_{\ell}|\sum_{d\in \mathcal{P}^{\text{s.a.}}} \mu(d) \lambda^{-\ell(d)} + \lambda^{\ell}\sum_{d\in \mathcal{P}^{\text{s.a.}}} \mu(d) \lambda^{-\ell(d)} \left(f(\ell-\ell(d))-f(\ell)\right). $$

We can now progress much further on making an additional assumption concerning the nature of the prime set $\mathcal {P}$. We could consider two possibilities: i) that $\mathcal {P}$ is the set of all primes on an induced subgraph *H*≺*G*; or ii) that $\mathcal {P}$ is a cut-off set, e.g. one disposes of all the primes of length *ℓ*(*γ*)≤*Θ*. Remarkably, in number theory, if i) is true then ii) is true as well, and the sieve benefits from the advantages of both situations. In general however, i) and ii) are not compatible and while ii) could be used to obtain direct estimates for the number of primes of any length, a problem of great interest, we can show that this makes the sieve NP-hard to implement. We therefore focus on the first situation.

Let *H*≺*G* be an induced subgraph of the graph *G* and let that $\mathcal {P}\equiv \mathcal {P}_{H}$ be the set of all primes (that is simple cycles) on *H*. Remark that $\sum _{d\in \mathcal {P}_{H}^{s.a}}\mu (d) \lambda ^{-\ell (d)}$ is therefore the sum over all the self-avoiding hikes on *H*, each with coefficient *μ*(*d*)*λ*^−*ℓ*(*d*)^. It follows (Giscard and Rochet 2017) that $\sum _{d\in \mathcal {P}_{H}^{s.a}}\mu (d) \lambda ^{-\ell (d)}=\det (\mathsf {I}-\lambda ^{-1}\mathsf {A}_{H})$. Concerning the error term,

$$\lambda^{\ell}\sum_{d\in \mathcal{P}_{H}^{\text{s.a.}}} \mu(d) \lambda^{-\ell(d)} \left(f(\ell-\ell(d))-f(\ell)\right), $$

we note that since *H* is finite,^3^ the above sum involves finitely many self-avoiding hikes *d*. In addition, given that ${\lim }_{\ell \to \infty }f(\ell)$ exists by Lemma 1, ${\lim }_{\ell \to \infty }f(\ell -\ell (d))-f(\ell) =0$ as long as *ℓ*(*d*) is finite, which is guaranteed by the finiteness of *H*. We have consequently established that the error term comprises finitely many terms, each of which vanishes in the *ℓ*→*∞* limit. As a corollary, the first term becomes asymptotically dominant:

$$S(\mathcal{H}_{\ell},\mathcal{P}) \sim |\mathcal{H}_{\ell}|\det\left(\mathsf{I}-\lambda^{-1}\mathsf{A}_{H}\right) ~~\text{as}~~\ell\to\infty. $$

We can make this more explicit on using the ordinary form of the zeta function on hikes *ζ*(*z*)=1/ det(I−*z*A). Then $|\mathcal {H}_{\ell }|=\zeta (z)[\ell ]$ is the coefficient of order *ℓ* in *ζ*(*z*), see also the proof of Lemma 1.

### Remark 1.

The error term can be given a determinantal form upon using a finite difference expansion of *f* or a Taylor series expansion of it if one smoothly extends its domain from $\mathbb {N}$ to $\mathbb {R}$. Writing

$$f(\ell-\ell(d))-f(\ell) = \sum_{k\geq 1} \frac{\nabla^{k}[f](\ell)}{k!}\left(\ell(d)\right)_{(k)}, $$

with $(a)_{(k)}=\prod _{i=0}^{k-1}(a-i)$ the falling factorial and ∇ the backward difference operator. Now we use the properties of the Möbius function on hikes to write $\sum _{d\in \mathcal {P}_{H}^{s.a}}\mu (d)\left (\ell (d)\right)_{(k)} \,z^{\ell (d)}= (\frac {d}{dz})^{k} \det (\mathsf {I}-z\mathsf {A}_{H})$ and finally

$$\begin{array}{*{20}l} S(\mathcal{H}_{\ell},\mathcal{P}) &= |\mathcal{H}_{\ell}|\,\det\!\left(\mathsf{I}-\frac{1}{\lambda}\mathsf{A}_{H}\right)+\lambda^{\ell}\sum_{k\geq 1}\frac{\nabla^{k}[f](\ell)}{k!} \det\!^{(k)}\!\left(\mathsf{I}-\frac{1}{\lambda}\mathsf{A}_{H}\right).\\[-1em] \end{array} $$

Here $\det ^{(k)}\left (\mathsf {I}-\frac {1}{\lambda }\mathsf {A}_{H}\right)$ is a short-hand notation for $\left \{(\frac {d}{dz})^{k} \det (\mathsf {I}-z\mathsf {A}_{H})\right \} \left |{~}_{z=\lambda ^{-1}}\right.$.

To conclude the proof of the Theorem, we now need only choose *H* correctly. Recall that we seek to count those walks which are left-multiples of a chosen simple cycle *γ*. But for *w*=*hγ* to be a walk, the hike *h* must be such that none of its right-prime divisor commutes with *γ*. This way, *γ* is guaranteed to be the unique prime that can be put to the right of *h*, hence the unique right-prime divisor of *w*, making *w* a walk. Then the sieve must eliminate all hikes *h* with are left-multiples of primes *commuting* with *γ*. Observe that all such primes are on *H*=*G*∖*γ*.

### Remark 2.

The construction presented here is much more general than appears at first glance. In particular, it can be extended to any additive function $\rho :\,\mathcal {H}\mapsto \mathbb {R}$ over $\mathcal {H}$ other than the length, provided an equivalent of Lemma 1 exists for *ρ*. Infinite graphs may also be considered, provided additional constraints on the notion of determinant are met. These generalisations have further applications which will be presented elsewhere.

## Comparison with Everett and Borgatti’s group-centralities

### Motivations and context

In our previous work on the centrality *c*(*H*) (Giscard and Wilson 2017b), we have presented comparisons with centralities obtained for *H* upon summing up the vertex centralities of individual vertices involved in *H*. We have shown the comparative failure of these approaches which could not, for example, detect even the major crisis affecting the insurance −*finance*−real-estate triad in input-output networks over the period 2000-2014 period.

In this section, we propose to further compare *c*(*H*) with the notion of group centrality as it was introduced by Everett and Borgatti in 1999 (Everett and Borgatti 1999). The authors of this study proposed to extend any vertex centrality to groups of vertices by summing up the centrality of the vertices of the group as calculated on a graph where other members of the group have been deleted. For example, the degree group centrality of an ensemble *H* of vertices is equal to the external degree of *H* in *G*. Essentially, this approach is expected to characterise the importance of the group with respect to the rest of the graph but will not be sensitive to the inner structure of the group. As a consequence, it is easy to construct synthetic graphs where group-centralities ’fail’ to identify a group that should clearly be the most central. For example, a sparse graph with a single large clique can be built such that this clique is less central than a small outlier group of nodes. In our opinion however, these limitations are more theoretical than practical and it is much more important to study the behaviour of the proposed measures on *real-world* networks.

### The centrality *c*(.) as an extension of the eigenvector centrality

Incidentally, Everett and Borgatti provide a strong motivation for the development of a centrality akin to the one we propose here. Indeed, noting the lack of extension for the eigenvector centrality to groups of nodes in their work, they explain that “[The eigenvector centrality] is virtually impossible to generalise along the lines presented earlier", that is, lest one resorts to node-merging, a procedure not without problems (Everett and Borgatti 1999). Now recall that the centrality presented here *c*(*H*) induces the eigenvector centrality on singleton subgraphs comprising exactly one vertex *H*={*i*}, a requirement which, following Everett and Borgatti, is sufficient to call *c*(.) a proper extension of the eigenvector centrality to groups of nodes. In fact, this observation is itself a special case of a more general construction relating the centrality of simple paths with entries of the projector onto the dominant eigenvector:

#### Proposition 1.

Let *G* be a finite undirected graph with {*λ*≡*λ*_1_,*λ*_2_,⋯,*λ*_*N*_} its spectrum. For simplicity, we assume that the largest eigenvalue *λ* of *G* is unique. Let $W:\mathcal {E}\mapsto \mathbb {R}^{+}$ be the weight function, sending edges of the graph to their weights. If *G* is not weighted then *W* is identically 1. Let P_*λ*_ be the projector onto the dominant eigenvector of *G* and $\eta :=\prod _{i>1}^{N}(1-\lambda _{i}/\lambda)$. Then

$$\eta(P_{\lambda})_{ij} =\sum_{p:\,i\to j} \lambda^{-\ell(p)}W(p)\,c(p), $$

where the sum runs over all simple paths from *i* to *j* and the weight of a path is the product of the weights of the edge it traverses.

#### Remark 3.

When *i*≡*j*, the only simple path from *i* to itself is the length 0 path that is stationary on *i*. The weight of the empty path is the empty product with value 1 and therefore we recover the result of (Giscard and Wilson 2017b)

$$\eta eig(i)^{2}=\eta \left(\mathsf{P}_{\lambda}\right)_{ii} = c(\{i\}), $$

where *eig*(*i*) is the *i*th entry of the dominant eigenvector.

#### *Proof*

This relation follows from e.g. the path-sum formulation of the resolvent function R(*z*):=(I−*z*A)^−1^ (Giscard et al. 2013). We have

$$\mathsf{R}(z)_{ij} =\sum_{p:\,i\to j} z^{\ell(p)}W(p)\,\frac{\det(\mathsf{I}-z\mathsf{A}_{G\backslash p})}{\det\left(\mathsf{I}-z\mathsf{A}\right)}. $$

In particular, the case *i*≡*j* gives the well-known adjugate formula for the inverse R(*z*)_*ii*_= det(I−*z*A_*G*∖*i*_)/ det(I−*z*A). Introducing the adjugate matrix Adj(I−*z*A)_*ij*_:= det(I−*z*A)R(*z*)_*ij*_ explicitly we have

$$\text{Adj}\left(\mathsf{I}-z\mathsf{A}\right)_{ij} =\sum_{p:\,i\to j} z^{\ell(p)}W(p)\,\det\left(\mathsf{I}-z\mathsf{A}_{G\backslash p}\right), $$

and the result follows on noting that ${\lim }_{z\to 1/\lambda }\text {Adj}\left (\mathsf {I}-z\mathsf {A}\right) = \eta \mathsf {P}_{\lambda }$. □

We can go further to establish the centrality *c*(.) as an extension of the eigenvector centrality to groups of nodes along broadly similar lines as those advocated by Everett and Borgatti. To introduce the main result here, we need to present the (intuitive) definitions of union and intersection of subgraphs.

Let *H*, *H*^′^ be two subgraphs of *G*. We designate by *H*∪*H*^′^ the subgraph of *G* whose vertex set is the set-theoretic union of the vertex sets of *H* and *H*^′^, $\mathcal {V}(H\cup {H^{\prime }})=\mathcal {V}(H)\cup \mathcal {V}({H^{\prime }})$. Similarly *H*∩*H*^′^ is the subgraph of *G* with vertex set $\mathcal {V}(H)\cap \mathcal {V}({H^{\prime }})$.

#### Proposition 2.

Let *G* be a finite graph with no negative weights and {*H*_1_,⋯*H*_*n*_} be a set of connected induced subgraphs of *G*. Then

$$c\left(\bigcup_{i=1}^{n}H_{i}\right)=\sum_{S\subseteq\{1,\cdots,n\}}(-1)^{|S|-1} c\left(\bigcap_{s\in S} H_{s}\right). $$

#### *Proof*

This follows from the definition of *c*(*H*) as the fraction of all network flows intercepted by *H*. A direct application of the inclusion-exclusion principle gives the result. □

An immediate corollary then explicitly shows how the centrality *c*(.) of any group of nodes arises from the interplay between their eigenvector centralities

#### Corollary 1.

Let *G* be a finite graph with no negative weights. Let $\mathcal {V}_{H}:=\{v_{1},\cdots,v_{n}\}\subseteq \mathcal {V}$ be a group of nodes on *G*. Then

$$c\left(\{v_{1},\cdots,v_{n}\}\right)=\eta\,\sum_{i=1}^{n} eig(v_{i})^{2} - \sum_{i,j\in \mathcal{V}_{H}}f\left(\{v_{i},v_{j}\}\right)+ \sum_{i,j,k\in \mathcal{V}_{H}}f\left(\{v_{i},v_{j},v_{k}\}\right)-\cdots, $$

where *f*({*v*_*i*_,*v*_*j*_,*v*_*k*_,⋯ }) is the fraction of all network flows intercepted by all of *v*_*i*_, *v*_*j*_, *v*_*k*_, etc.

### Wolfe’s dataset

We begin our concrete comparison with group-centralities on the Wolfe primate dataset (UCINET IV Datasets 2018), a small real-world network which was studied by Everett and Borgatti. This dataset provides the number of times monkeys of a group of 20 have been spotted together next to a river by the anthropologist Linda Wolfe.

Our results are shown in Table 1. Here the properties that *c* is always between 0 and 1 and that its values have actual meaning are clearly advantageous. For example, we can now not only tell that the age group 10 −13 is the most central, as Everett and Borgatti noted, but we can concretely assert that 67% of all flows of interactions between monkeys involved at least one member of this group. By flow (or chain) of interactions, we mean successions of interactions between monkeys, including interactions that may occur simultaneously. For example, we can have monkey 1 interact with 3, who then interacts with 8; while concurrently 2 meets 4 etc.

**Table 1:** Comparison between several of Everett and Borgatti’s group centralities (Everett and Borgatti 1999) and the centrality *c*(*H*). The centrality values for *c*(*H*) are given here in % as they give the proportions of all successions of interactions between monkeys involving at least one member of the group. The centralities *c*(*H*) were computed by the FlowFraction algorithm available on the Matlab File Exchange (Giscard and Wilson 2017a)

	Centralities of groups of monkeys in Wolfe’s dataset				
Group	Members	*c*(*H*)in %	Degree	Average closeness	Group
			group centrality	group centrality	betweenness
Age 10 −13	2 3 8 12 16	67%	11	15	43.5
Age 7 −9	4 5 9 10 15 17	57%	5	13.7	0
Age 14 −16	1 6 11 13 19	49%	8	18	2.84
Age 4 −6	7 14 18 20	34%	5	20.5	0
Females	6−20	95%	4	6.4	0.5
Males	1−5	64%	10	16	24.34

Similarly, we note that almost 95% of all flows of interactions involved at least one female, while this percentage dropped to 64% for males, in spite of male 3 being the most central individual monkey in the entire group by all measures. Thus, according to *c*(*H*) and contrary to all the group centralities reported here,^4^ females are quantitatively more important in mediating social interactions than the males. Here, it may help to know that the monkeys observed by Wolfe were feral Rhesus macaques (*Macaca mulatta*), a species where females stay in the group of their birth, providing its dominance rank structure, while males must change group when reaching sexual maturity, around 4 years old. Furthermore, during the mating season, females favour multiples interactions with different males including low ranking ones (Lindburg 1971). Finally, females typically outnumber males, sometimes by as much as 3 to 1. These observations suggest that females should indeed account for a larger share of the all interactions between monkeys than the males.

Another point of importance for the comparison is the age group 7 −9, which is ranked higher than the age group 14 −16 by *c*(*H*) while the group-centralities consistently yield the opposite order. On this point, we observe that Rhesus macaques are peculiar in that younger females have higher social ranks than their older peers (Hill and Okayasu 1996;Wall 1993). In the closely related Japanese macaques (*Macaca fuscata*), dominance rank is known to be positively correlated with the frequency of social interactions (Singh et al. 1996).

### Yeast PPI network and protein complexes

In this section we study the PPI network of the yeast *Saccharomyces cerevisiae*, using high quality data from (Hart et al. 2007), which provides a network comprising 5303 interactions between 1689 individual proteins. These proteins are known to belong to complexes, a curated list of which is provided by the Munich Information center on Protein Sequences (MIPS) (Güldener et al. 2006). The authors of (Hart et al. 2007) have shown that some of the MIPS complexes could be recovered from a run of the MCL clustering algorithm running on the network. Our goal here is twofold: i) to show that the centrality *c*(.) can also be used to recover MIPS protein complexes, for which it provides additional informations; and ii) that the degree group centrality fails to do so. Here, we focus specifically on the degree group centrality as the degree centrality is the vertex measure of importance which has seen the most success in biology, see e.g. (Mukhtar and et al 2011).

#### Analysis

**Fig. 2:**
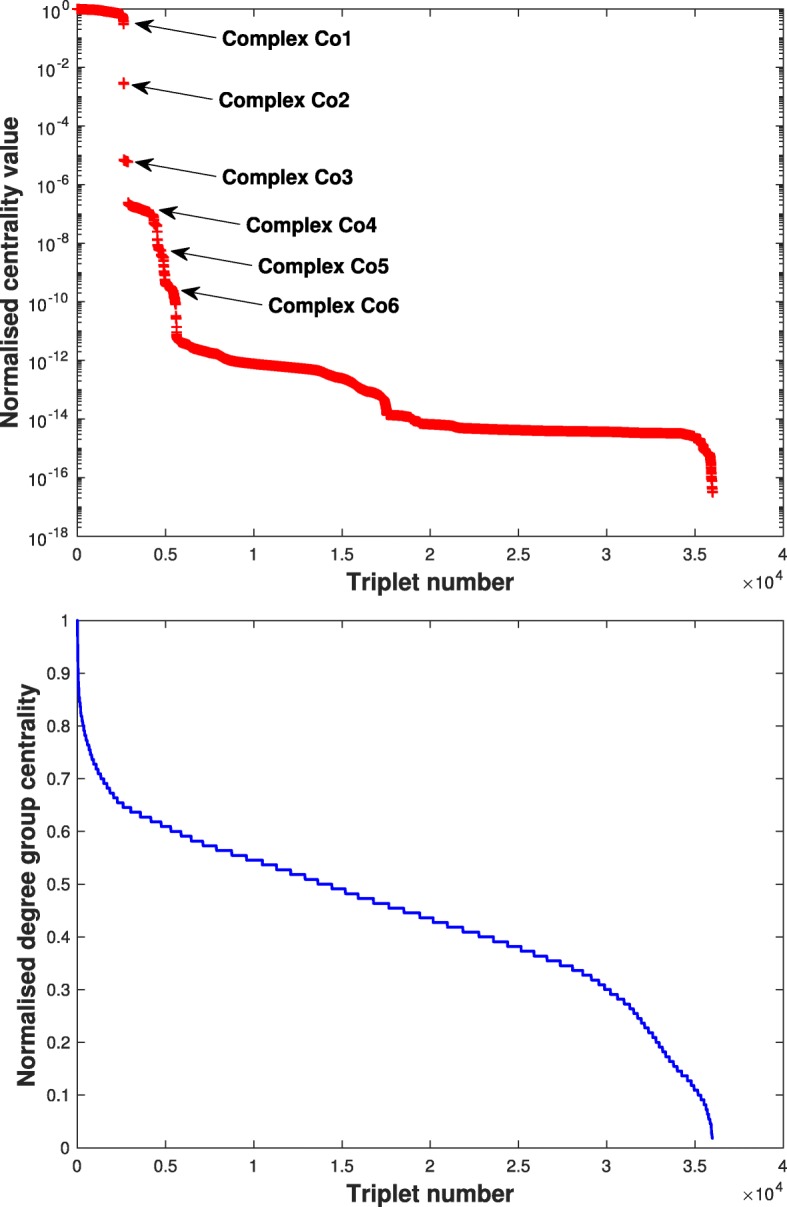
Distributions of triplet centralities. Top: normalised triplet centralities *c*(*t*)/ max*t* triplet(*c*(*t*)), bottom: normalised degree group centrality *g*(*t*)/ max*t* triplet(*g*(*t*)) introduced in (Everett and Borgatti 1999)

We analysed the PPI in three steps. Firstly, we found all edges (that is connected pairs of vertices) connected triplets (triangles and paths on 3 vertices) and connected quadruplets of proteins on the network.^5^ Secondly, we calculated the centralities *c*(.) of these objects. To present the third step of our analysis, we invite the reader to observe the distribution of centrality values, which we show at the top of Fig. 2 in the case of triplets.^6^ Clearly, high triplet centralities fall into separate plateau-like ensembles. Therefore, the third and final step of our analysis is to gather the list of all proteins appearing in all the triplets whose centrality values placed them in the same plateau. We then compare these lists of proteins with the biological complexes found in curated databases (Pu et al. 2009). Remarkably, these lists of proteins correspond to actual biological complexes. We summarise our observations as follows :

- Plateaus, i.e. groups of triplets with similar centrality values, correspond to actual protein complexes;
- Conversely, all triplets belonging to an actual complex are in the same plateau, i.e. they scored roughly the same in centrality values;
- Consider a triplet *t* whose centrality value *c*(*t*) is in one of the top plateaus. Then all three proteins forming *t* are part of the same biological complex.
- Triplets with small centrality values, outside of plateaus, tend to belong to no particular complex or several complexes at once (i.e. one protein in one complex, the other two in another).

We emphasise that in our analysis the complexes are determined entirely from the plateaus of centrality values. That these so-determined complexes correspond to actual biological complexes demonstrates the quality of the analysis provided by the centrality proposed here.

Mathematically, the fact that biological complexes lead to clustered plateau-like centrality values for triplets means that the frequency with which proteins belonging to these complexes are involved in successions of proteins reactions depends first and foremost on the complexes themselves. In other terms, the frequency of protein activation is determined at the complex level.

#### Identified complexes

The dominant complex, here denoted Co1, comprises 30 proteins^7^ and is found in both the MIPS database and in (Pu et al. 2009), where it is known as the mitochondrial small ribosomal large subunit. Interestingly, Co1 is identical with the third largest complex recovered by the MCL algorithm running on the same dataset (Hart et al. 2007), with the addition of the proteins ASF1 and MAM33, a nucleosome assembly factor and a protein of the mitochondrial matrix involved in oxidative phosphorylation, respectively. In the latter case, we note that several complexes involving the MAM33 and proteins of mitochondrial small ribosomal large subunit have been proposed in experimental studies (Yeast Resource Center 2018). Complex Co2 comprises 21 proteins.^8^ It includes the entire complex C17 determined by the MCL method (Hart et al. 2007), together with 6 additional proteins all which are been proposed to form complexes (in particular the HIR and Rad53p-Asf1p complexes) with one or more proteins of C17 in separate studies (Pu et al. 2009) as well as in the MIPS database. Complex Co3 comprises 64 proteins and overlaps significantly with the nucleosomal protein and CID 14 and complexes of (Pu et al. 2009), the latter of which includes the Casein kinase II, RNA polymerase II and Cdc73/Paf1 complexes.^9^

An advantage of the classification method employed here is that, contrary to MCL, it allows for overlapping complexes, i.e. proteins which functions in different complexes, as is expected biologically. At the same time, a drawback is that small centrality values are not segregated well enough to clearly distinguish clusters of values and hence complex boundaries. At least three more complexes Co4, Co5 and Co6 could possibly be distinguished, all of which can be found in MIPS database, however these are less clear cut than the first three complexes and so are left out from this work. Empirically, we found that this problem could be somewhat reduced by looking at quadruplets, quintuplets etc., but this comes at a great computational cost given the number of such objects. A random sampling scheme may be able to bypass this difficulty.

In comparison, the distribution of degree group centrality shows no trace of the underlying protein complexes and reveals little more than the simple distribution of vertex degrees. While we do not recommend the use of the centrality *c*(.) as a clustering tool owing to its greater computational cost than algorithms such as MCL, we believe that its performance in this domain bears witness to the sensitivity of the proposed centrality to underlying network features. Conversely, the notion of group-centrality may be too coarse to perceived such features in the data, at least in the case of PPI.

## Conclusion

In this second work on the centrality *c*(.), we have rigorously established its meaning as a fraction of network flows intercepted by any chosen ensembles of nodes. The centrality *c*(.) not only induces the eigenvector centrality on vertices, but it is a proper extension of it through an application of the inclusion-exclusion principle on network flows. Finally, we have shown on two real-world networks that the centrality *c*(.) is more sensitive to critical network features than existing group-centralities. In particular, the centrality of triplets of proteins in the PPI network of the yeast was sufficient to distinguish protein complexes found in curated databases of experimental results. We recall that in our previous study (Giscard and Wilson 2017b), the centrality *c*(.) already produced the best available model for pathogen targeting in *Arabidopsis thaliana*, yielding a 25% improvement of the state-of-the-art model of (Mukhtar and et al 2011). We hope that these results will spur further research on the use of the centrality in biology.

## Footnotes

1 We refer to the area under the ROC curves for both the model based on the centrality *c*(.) and the state of the art one. These are 0.97 and 0.73 respectively.

2 But not necessarily totally multiplicative.

3 *G* is finite and so are all its induced subgraphs.

4 Everett and Borgatti also discuss normalisations of the group-centralities. In the case of the degree group-centrality, the normalisation is defined to be the degree group centrality divided by the number of nodes which do not belong to the group under consideration. Normalisations tends to rank females ahead of males as *c*(*H*) does, but they represent non-linear transformation of the original group-centralities, making their interpretation more difficult.

5 While the subgraph centrality is defined on arbitrary subgraphs, including non-connected ones, here we only consider only connected ensembles of proteins as these may have biological activities.

6 Edges and quadruplets give broadly similar distributions. While complexes Co1, Co2 and Co3 are just as markedly visible in quadruplet data as in triplet data, quadruplets do lead to better segregation of complexes Co4, Co5 and Co6.

7 It comprises proteins ASF1, EHD3, FYV4, MAM33, MRP1, MRP4, MRP10, MRP13, MRP21, MRP51, MRPS5, MRPS8, MRPS9, MRPS16, MRPS17, MRPS18, MRPS28, MRPS35, NAM9, PET123, RSM7, RSM10, RSM18, RSM19, RSM22, RSM23, RSM24, RSM25, RSM26 and RSM27.

8 These are ASF1, CDC48, CKA1, HAT1, HAT2, HHF1, HHF2, HHT2, HIF1, HIR2, PDS5, POB3, PSE1, PSH1, RAD53, RTG2, RTT106, SPT16, YDL156W, YIL070C and YKU70.

9 This complex is ASF1, CDC34, CDC48, CDC53, CDC73, CDC9, CHD1, CKA1, CKA2, CKB1, CKB2, CTR9, DOA1, GRR1, HAT1, HAT2, HHF1, HHF2, HHT2, HIF1, HIR1, HIR2, HOT1, HPC2, HTA1, KAP114, LEO1, MET30, MKT1, MRF1, NAP1, NPL4, ORC2, ORC3, ORC4, ORC5, PAF1, PDS5, PEX19, POB3, POL12, PSE1, PSH1, RAD27, RAD53, RPS1B, RRP7, RTF1, RTG2, RTT101, RTT106, SHP1, SKP1, SPO12, SPT16, TOP1, UFD1, ULP1, UTP22, YDL156W, YDR049W, YGR017W, YKU70 and YKU80
